# MemRoadNet: Human-like Memory Integration for Free Road Space Detection

**DOI:** 10.3390/s25216600

**Published:** 2025-10-27

**Authors:** Sidra Shafiq, Abdullah Aman Khan, Jie Shao

**Affiliations:** 1 School of Computer Science and Engineering, University of Electronic Science and Technology of China, Chengdu 611731, China; sidra@std.uestc.edu.cn (S.S.); abdkhan@uestc.edu.cn (A.A.K.); 2 Sichuan Artificial Intelligence Research Institute, Yibin 644000, China

**Keywords:** road segmentation, representation learning, feature matching, autonomous driving, image feature extraction

## Abstract

Detecting available road space is a fundamental task for autonomous driving vehicles, requiring robust image feature extraction methods that operate reliably across diverse sensor-captured scenarios. However, existing approaches process each input independently without leveraging Accumulated Experiential Knowledge (AEK), limiting their adaptability and reliability. In order to explore the impact of AEK, we introduce MemRoadNet, a Memory-Augmented (MA) semantic segmentation framework that integrates human-inspired cognitive architectures with deep-learning models for free road space detection. Our approach combines an InternImage-XL backbone with a UPerNet decoder and a Human-like Memory Bank system implementing episodic, semantic, and working memory subsystems. The memory system stores road experiences with emotional valences based on segmentation performance, enabling intelligent retrieval and integration of relevant historical patterns during training and inference. Experimental validation on the KITTI road, Cityscapes, and R2D benchmarks demonstrates that our single-modality RGB approach achieves competitive performance with complex multimodal systems while maintaining computational efficiency and achieving top performance among single-modality methods. The MA framework represents a significant advancement in sensor-based computer vision systems, bridging computational efficiency and segmentation quality for autonomous driving applications.

## 1. Introduction

As sensor technology continues to advance and integrate with computer vision systems, the development of efficient image feature extraction methods becomes increasingly crucial. These methods are essential for applications across various domains, including autonomous driving, smart cities, and intelligent transportation systems. Such applications require robust models capable of accurate drivable region detection across diverse environmental conditions [1]. The precision of road detection/segmentation directly influences navigation safety, path planning efficiency, and overall autonomous vehicle performance in real-world scenarios. Contemporary deep-learning approaches, particularly Convolutional Neural Networks (CNN), have demonstrated substantial progress [2] in addressing this perception task. They achieve this through hierarchical feature learning and multi-scale spatial reasoning [3,4].

Despite these advances, current methodologies face several limitations that constrain their practical effectiveness. Traditional CNN architectures [5] process each input independently. Such architectures do not leverage accumulated experiences or contextual knowledge from previously encountered scenarios. This approach becomes particularly problematic in dynamic driving environments where road conditions, lighting variations, weather patterns, and infrastructure configurations exhibit significant diversity across geographical regions and temporal contexts [6]. While achieving impressive accuracy benchmarks, these models often require substantial computational resources that may not be readily available in embedded vehicle systems [7]. Additionally, most current approaches lack mechanisms to learn from both successful and unsuccessful prediction attempts, and this limitation causes them to miss opportunities to build comprehensive knowledge bases that could inform future segmentation decisions.

Recent advances in Memory-Augmented (MA) neural networks [8,9] have demonstrated promising potential for addressing these fundamental limitations. Drawing inspiration from human cognitive processes, researchers have begun exploring neural architectures that incorporate persistent memory mechanisms. Human cognitive processes effectively leverage episodic and semantic memory systems to inform current decision-making. These new architectures aim for enhanced pattern recognition and contextual reasoning. However, the application of MA approaches to semantic segmentation tasks, particularly free road space detection, remains largely unexplored. Most contemporary approaches rely on multimodal sensor fusion or complex ensemble architectures to achieve competitive performance, increasing computational overhead and system complexity.

For this purpose, we introduce a Human-like Memory Bank that draws inspiration from cognitive neuroscience theories of human memory systems [10,11], while implementing these concepts through established deep-learning mechanisms. The episodic memory module uses similarity-based retrieval to store and recall specific driving experiences, analogous to how humans retrieve context-specific memories. The working memory employs attention mechanisms to maintain task-relevant information during inference. While these modules use conventional machine learning techniques (attention, similarity metrics, weighting), their architectural organization and interaction patterns are inspired by cognitive models of human memory. The emotional valence mechanism implements a quality-based weighting system that prioritizes high-quality experiences, drawing from theories of emotional memory consolidation [10,11]. We emphasize that these are engineering implementations inspired by neuroscience concepts, rather than direct computational models of neural processes.

The memory system implements three interconnected subsystems: episodic memory for storing specific experiences, semantic memory for maintaining generalized knowledge patterns, and working memory for preserving recent contextual information. The MA architecture enables learning from both successful and unsuccessful prediction attempts. This builds a comprehensive knowledge base that informs future segmentation decisions. We implement memory consolidation and forgetting mechanisms that prioritize important experiences. The framework employs contextual encoding that captures visual, spatial, and performance-related information, and this enables rich associative memory retrieval based on current scene characteristics.

Motivated by these observations, this paper addresses the fundamental gap in how neural networks can accumulate and utilize experiential learning for improved free road space detection. We propose a framework that bridges this critical gap by integrating accumulated experiences to enhance current prediction accuracy while operating exclusively on single-modality RGB input. Despite this computational constraint, our approach achieves performance comparable to sophisticated multimodal systems through intelligent memory utilization. The framework combines an InternImage-XL backbone [12] with a UPerNet decoder architecture (similar to [13]), augmented by a Human-like Memory Bank system inspired by cognitive neuroscience principles.

The system extracts compressed feature representations from deep network activations, associates them with contextual information, including visual statistics and performance outcomes, and stores these experiences with emotional valence based on prediction accuracy. Memory retrieval employs similarity computation considering pattern alignment, contextual relevance, temporal recency, and learned importance scores to identify relevant past experiences for current prediction tasks. The integration of retrieved memories with current network processing occurs through attention-based mechanisms that enable selective incorporation of relevant historical knowledge while preserving the network’s ability to process novel scenarios [14].

Experimental validation on challenging road segmentation benchmarks demonstrates the effectiveness of our approach. The framework achieves superior performance compared with single-modality-based methods and competitive results with multimodal approaches. We conduct comprehensive ablation studies to reveal the individual contributions of different memory subsystems and provide insights into memory dynamics that drive performance improvements. The primary contributions of this work can be summarized as follows:

- We present a framework that integrates human-inspired cognitive architectures implementing episodic, semantic, and working memory subsystems with biologically inspired consolidation and forgetting mechanisms for enhanced performance.
- Our comprehensive experiments demonstrate superior performance among state-of-the-art single-modality-based methods and competitive performance approaching multimodal systems on challenging road segmentation benchmarks. Additionally, we present a detailed analysis of memory dynamics, retrieval mechanisms, and their impact on performance.

The rest of this paper is organized as follows. Section 2 reviews related work in semantic segmentation, MA neural networks, and cognitive architectures. Section 3 presents our framework, including the InternImage-XL backbone, UPerNet decoder, and Human-like Memory Bank system. Section 4 provides comprehensive experimental validation and ablation studies. Section 5 discusses limitations, environmental impact considerations, and future research directions. Section 6 concludes with a discussion of results and future research directions. The code, weights, and other materials can be found at our GitHub webpage (https://github.com/abdkhanstd/MemRoadNet, accessed on 19 October 2025).

## 2. Related Work

Free road space detection has witnessed substantial advancement through deep-learning methodologies. It spans both complex multimodal frameworks and computationally efficient single-modality approaches. However, existing architectures fundamentally lack mechanisms for leveraging Accumulated Experiential Knowledge (AEK), processing each input independently without benefiting from previously encountered patterns or contextual associations.

### 2.1. Multimodal Approaches

Modern multimodal methods demonstrate remarkable performance through sophisticated sensor fusion strategies, integrating complementary information from LiDAR, RGB imagery, depth sensors, and surface normal estimations. For example, Evi-RoadSeg [15] presents an evidence-based approach for real-time road segmentation, enhancing performance through RGB-D data augmentation. Similarly, the study named LFD-RoadSeg [16] introduces a method for ultra-fast road segmentation by leveraging low-level representations to enhance efficiency and performance. Moreover, CLCFNet [17] implements cascaded LiDAR-camera processing pipelines, while PLARD [18] achieves state-of-the-art performance through adaptive LiDAR data integration. USNet [19] delivers low-latency processing via lightweight symmetric network architectures, addressing real-time deployment requirements for autonomous systems.

Surface normal estimation methodologies, including SNE-RoadSeg [20] and its enhanced variant SNE-RoadSegV2 [21], advance heterogeneous feature fusion through fallibility-aware processing mechanisms. Recent depth-aware approaches have further expanded multimodal capabilities. For example, the research named DiPFormer [22] explores deep RGB-D interactions for traffic scene segmentation, demonstrating how depth information enhances spatial understanding in complex driving environments. Transformer-based approaches such as RoadFormer [23] and RoadFormer+ [24] employ scale-aware information decoupling for RGB-Normal semantic parsing, showcasing attention mechanisms’ effectiveness in multimodal integration.

Additional multimodal frameworks, including DFM-RTFNet [25], 3MT-RoadSeg [26], Pseudo-LiDAR [27], TEDNet [28], CLRD [29], and LRDNet [14], present diverse multimodal integration strategies, each addressing specific challenges in sensor fusion and computational efficiency trade-offs. Contemporary approaches have expanded to include LiDAR-image fusion methodologies, exemplified by UdeerLID+ [30], which integrates LiDAR, image, and relative depth information through semi-supervised learning paradigms. Furthermore, pseudo-LiDAR techniques have emerged as cost-effective alternatives, with [27] demonstrating effective road detection through depth-derived LiDAR representations, bridging the gap between expensive sensor suites and accessible RGB-based systems.

### 2.2. Methods Based on Single-Modality

Single-modality approaches prioritize computational efficiency while maintaining competitive performance through architectural innovations and algorithmic optimizations. LC-CRF [31] leverages conditional random field frameworks for structured prediction, while LFD-RoadSeg [16] employs bilateral network structures for enhanced feature representation. Additionally, specialized resource-efficient architectures, including RoadNet3 [32], ChipNet [33], DEEP-DIG [34], HA-DeepLabv3+ [35], RBANet [36], and Hadamard-FCN [37] demonstrate various strategies for reducing computational overhead while preserving segmentation accuracy. These approaches typically focus on architectural efficiency through lightweight convolutions, parameter reduction, or specialized design patterns.

### 2.3. Research Gap

Despite substantial progress in both multimodal and single-modality road segmentation, existing methodologies share a fundamental limitation, i.e., the inability to leverage AEK for enhanced prediction accuracy. Current architectures process each input independently, failing to capitalize on patterns, contextual associations, and performance feedback accumulated during training and deployment. Furthermore, while multimodal approaches achieve superior performance, they require complex sensor suites and substantial computational resources that may not be practical for all deployment scenarios. Single-modality methods, though more efficient, typically sacrifice performance to achieve computational constraints. Thus, this paper addresses these limitations by introducing MA mechanisms that enable single-modality approaches to achieve performance comparable with multimodal systems through intelligent utilization of AEK.

## 3. Methodology

**Figure 1:**
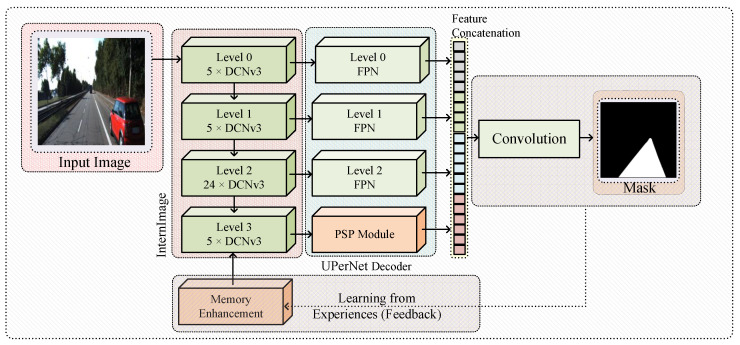
Simplified architectural overview showing the interaction of the InternImage-XL backbone (with DCNv3 blocks), UPerNet decoder (with FPN and PSP modules), and Memory Bank system.

This section presents our semantic segmentation framework that integrates human-inspired cognitive architectures. Figure 1 provides a simplified overview of the complete framework, illustrating how features flow from the backbone through the decoder while being enhanced by accumulated memory experiences.

### 3.1. Overall Architecture

**Figure 2:**
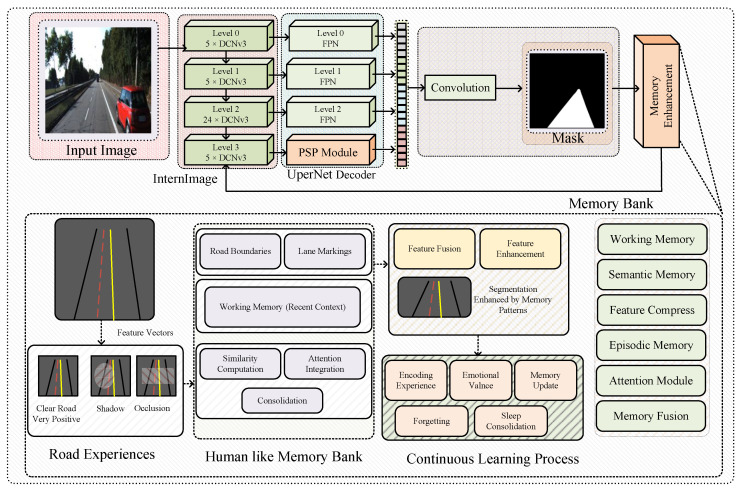
Comprehensive overview of our framework illustrating both architectural components and experiential learning processes. The upper panel demonstrates the technical integration of InternImage (XL) backbone, UPerNet decoder, and Human-like Memory Bank. The lower part demonstrates the memory system’s internal mechanisms, showcasing actual road experience categorization with emotional valences ranging from very positive (clear road scenarios) to negative (occluded conditions), episodic memory organization, semantic clustering, and the continuous learning feedback loop that enables experiential knowledge accumulation throughout training and inference phases.

The proposed framework orchestrates three interconnected components as illustrated in Figure 2, i.e., an InternImage-XL backbone for hierarchical feature extraction, a UPerNet decoder for multi-scale feature fusion, and our human-inspired Memory Bank that enables experiential learning through accumulated knowledge. Figure 2 shows the memory system’s operational dynamics. It shows how specific road experiences, ranging from clear highway segments to challenging shadowed intersections, are encoded with emotional valences based on segmentation performance outcomes. This detailed representation demonstrates the categorization mechanisms that enable our framework to distinguish between highly successful predictions (very positive emotional valence) and problematic scenarios (negative valence). This attempts to create a rich experience repository that fundamentally transforms how neural networks approach road segmentation tasks. The framework operates through an interplay between perception and memory, formulated as:

(1) $$\hat{Y}=\Omega \left(\Psi_{\mathrm{decoder}}\left(\Phi_{\mathrm{backbone}}\left(X\right)⊕R_{M}\left(X,C\left(X\right)\right)\right)\right),$$

where $X\in R^{B\times 3\times H\times W}$ represents input imagery, C(X) extracts contextual information, ⊕ denotes memory-guided feature enhancement, $R_{M}$ represents our memory recall function, and Ω applies sigmoid activation for binary road segmentation. The architecture’s novelty lies in its dual operational modes, i.e., during training and inference, the Memory Bank continuously accumulates experiential patterns from prediction outcomes and performance feedback, building a comprehensive repository of associations. During training and inference, this accumulated knowledge guides current predictions through intelligent retrieval and integration of relevant historical experiences.

### 3.2. InternImage-XL Backbone with DCNv3

The InternImage-XL backbone serves as our primary feature extraction mechanism, employing Deformable Convolution v3 (DCNv3) operations [38] for adaptive spatial modeling. This architectural choice stems from DCNv3’s superior capability in modeling long-range dependencies while capturing fine-grained spatial details through dynamic receptive field adaptation, making it particularly effective for complex segmentation scenarios where object boundaries exhibit significant geometric variation. As depicted in Figure 2, the backbone implements a hierarchical structure across four distinct levels, each engineered to capture features at different semantic granularities. The channel progression follows {192,384,768,1536} with corresponding spatial resolution reductions achieved through progressive downsampling operations. Each InternImage block/level (i.e., $B_{i}$) processes features through a sequence represented as:

(2) $$F_{i+1}=B_{i}\left(F_{i}\right)=F_{i}+\gamma_{1}\cdot \mathrm{DCNv}3\left(\Lambda_{1}\left(F_{i}\right)\right)+\gamma_{2}\cdot \mathrm{MLP}\left(\Lambda_{2}\left(F_{i}\right)\right),$$

where $F_{i}\in R^{C_{i}\times H_{i}\times W_{i}}$ represents features at level *i*, layer normalization operations $\Lambda_{1}$ and $\Lambda_{2}$ ensure stable training dynamics, and learnable layer scale parameters $\gamma_{1},\gamma_{2}\in R^{C_{i}}$ provide fine-grained control over feature integration. The DCNv3 operation forms the computational core of our backbone, adaptively sampling features based on learned offset and mask predictions:

(3) $$\mathrm{DCNv}3\left(F\right)=\sum_{k=1}^{K}w_{k}\cdot F\left(p_{0}+p_{k}+\Delta p_{k}\right)\cdot m_{k}\cdot \sigma \left(\alpha_{k}\right),$$

where $p_{0}$ represents the reference position, $\Delta p_{k}\in R^{2}$ represents learned spatial offsets enabling adaptive sampling, $m_{k}\in \left[0,1\right]$ represents attention masks modulating feature importance, $w_{k}$ denotes standard convolution weights, and $\sigma (\alpha_{k})$ represents learned modulation scalars. Level 3, as highlighted in our architecture diagram, receives particular attention as it serves dual purposes: providing the deepest semantic representations for decoder processing while simultaneously contributing to memory formation through feature compression operations. This dual utilization ensures that our memory system operates on the most semantically rich representations available from the backbone network.

### 3.3. UPerNet Decoder Head

The UPerNet decoder implements multi-scale feature fusion through its Feature Pyramid Network (FPN) [39] foundation, enhanced with Pyramid Scene Parsing (PSP) modules [40]. As illustrated in Figure 2, the decoder processes backbone features through distinct pathways: Levels 0-2 undergo lateral convolution followed by FPN refinement, while Level 3 (potentially enhanced by memory) passes through the PSP module for global context aggregation. The decoder establishes lateral connections that transform backbone features to unified channel dimensions:

(4) $$L_{i}=\Xi_{i}\left(F^{\left(i\right)}\right)+\Upsilon \left(L_{i+1}\right),$$

where lateral operations $\Xi_{i}$ implement 1×1 convolutions with batch normalization and ReLU activation, and the upsampling function Υ employs bilinear interpolation for spatial alignment across pyramid levels. The PSP module captures global contextual information through multi-scale pooling operations:

(5) $$\mathrm{PSP}\left(F\right)=\mathrm{Bottleneck}(\mathrm{Cat}\left(F,\underset{s\in \{1,2,3,6\}}{⋃}\Theta_{s}\left({\mathrm{Pool}}_{s}\left(F\right)\right)\right)),$$

where adaptive pooling operations ${\mathrm{Pool}}_{s}$ reduce spatial dimensions to s×s grids, projection functions $\Theta_{s}$ perform channel-wise transformations, and Cat accomplishes concatenation along the channel dimension. The feature concatenation stage, as shown in our architecture diagram, unifies all processed features through spatial alignment and channel concatenation before the final convolution operation produces the segmentation mask. This multi-scale integration ensures that both fine-grained local details and global contextual information contribute to the final prediction.

### 3.4. Human-like Memory Bank System

Our Memory Bank system is fundamentally different from traditional neural architectures. It incorporates persistent, adaptive memory mechanisms inspired by human cognitive processes. The system addresses the inherent limitation of feed-forward networks that process each input independently without benefiting from AEK or contextual associations developed through training.

#### 3.4.1. Memory Architecture Design

The Memory Bank, as shown in Figure 2, implements six interconnected components that collectively enable experiential learning. The system architecture comprises:

(6) $$\begin{array}{c} M=\{M_{\mathrm{working}},M_{\mathrm{episodic}},M_{\mathrm{semantic}},A_{\mathrm{attention}},\Sigma_{\mathrm{compress}},\Phi_{\mathrm{fusion}}\}. \end{array}$$

Working Memory maintains recent experiential context through a limited-capacity buffer. It is implemented as $M_{\mathrm{working}}=\mathrm{Deque}\left(\left\{e_{t-9},\ldots ,e_{t}\right\},\mathrm{maxlen}=10\right)$, ensuring immediate contextual information remains readily accessible for rapid integration with current processing. Furthermore, Episodic Memory serves as the primary repository for specific experiences. It stores comprehensive representations as tuples $e_{i}=\left(\phi_{i},\kappa_{i},\epsilon_{i},\tau_{i},\alpha_{i},\iota_{i}\right)$. Here, $\phi_{i}\in R^{128}$ represents compressed feature patterns, $\kappa_{i}$ encodes rich contextual information, $\epsilon_{i}$ captures emotional valence based on performance outcomes. Additional metadata tracks temporal and access characteristics. Semantic Memory organizes generalized knowledge patterns grouped by performance characteristics and emotional categories. This enables rapid access to successful strategies and pattern recognition approaches that have proven effective across diverse scenarios. The practical implementation of our memory architecture, as demonstrated in the detailed Memory Bank visualization of Figure 2, operates on concrete road experiences that exemplify the system’s categorization capabilities. Each stored experience represents a specific encounter with varying road conditions. These range from pristine highway segments that achieve very positive emotional valence through exceptional segmentation accuracy to challenging scenarios involving shadows, occlusions, or complex geometric configurations that receive correspondingly lower valence scores.

#### 3.4.2. Experience Encoding and Memory Formation

During training, our system continuously accumulates experiential knowledge through encoding mechanisms. Feature compression, as depicted in our architecture, transforms high-dimensional backbone activations into compact memory representations:

(7) $$\phi =\Sigma \left(\mathrm{GAP}\left(F^{\left(3\right)}\right)\right)=\mathrm{Linear}\left(\frac{1}{H_{3}W_{3}}\sum_{h=1}^{H_{3}}\sum_{w=1}^{W_{3}}F_{:,h,w}^{\left(3\right)}\right),$$

where $\Sigma :R^{1536}\to R^{128}$ represents a learned transformation that preserves semantic content through dimensionality reduction. Contextual encoding captures multimodal scene properties that provide rich environmental information for memory association:

(8) $$\kappa =\{\kappa_{\mathrm{visual}},\kappa_{\mathrm{spatial}},\kappa_{\mathrm{training}},\kappa_{\mathrm{performance}}\}.$$

Visual context encompasses statistical image properties, including brightness characteristics, contrast metrics, and per-channel statistics. Spatial context encodes geometric properties, while training context maintains meta-information regarding the current training state. Performance context captures prediction quality indicators when ground-truth annotations are available. Emotional valence quantifies experience significance through IoU (Intersection over Union)-based categorization: very positive (IoU >0.8), positive (0.6<IoU≤0.8), neutral (0.4<IoU≤0.6), negative (0.2<IoU≤0.4), and very negative (IoU ≤0.2). This enables prioritizing both highly successful experiences and significant failures as valuable learning signals.

#### 3.4.3. Memory Recall and Integration

The memory recall process, as illustrated through the concrete examples in Figure 2, demonstrates how our system leverages accumulated road experiences to inform current predictions. The episodic memory component maintains detailed records of specific encounters, clear road boundaries that achieved exceptional performance, challenging shadow patterns that required discrimination, and complex intersection geometries that tested the network’s spatial reasoning capabilities. This rich experiential foundation enables the attention module to identify relevant historical patterns that share semantic or contextual similarity with current input scenarios, creating intelligent associations that guide enhanced feature representations toward more accurate segmentation outcomes.

**Figure 3:**
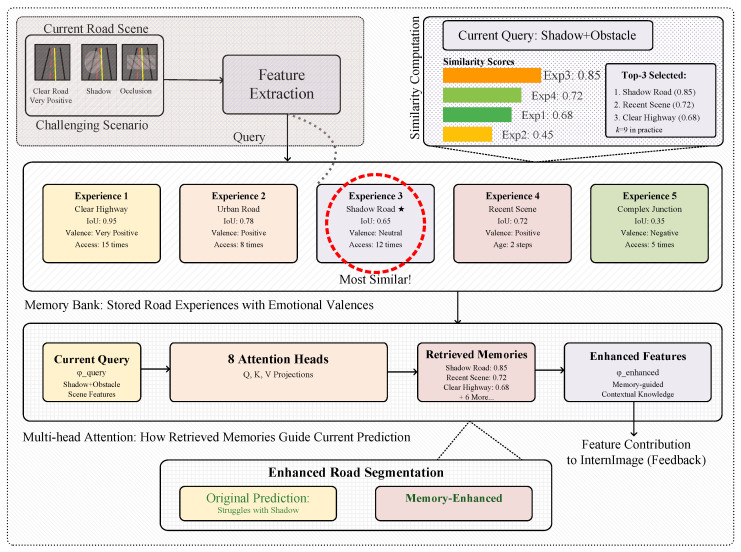
Detailed illustration of the memory usage, showing episodic memory retrieval, attention-based pattern matching, and memory-guided feature enhancement processes. ★ and the dashed red ellipse indicate most similar.

During training, the memory system transitions from accumulation mode to utilization mode, leveraging AEK to enhance current predictions. Figure 3 illustrates the detailed memory recall and integration processes that enable contextual reasoning during road segmentation tasks. The attention module, as shown in Figure 2, implements retrieval mechanisms that identify relevant historical experiences through multi-faceted similarity computation that extends beyond simple cosine similarity to incorporate human-like memory characteristics. The enhanced memory retrieval employs a comprehensive similarity metric that combines pattern matching, contextual relevance, temporal dynamics, and learned importance:

(9) $$s_{\mathrm{total}}\left(e_{i},q\right)=\alpha \cdot s_{\mathrm{pattern}}\left(e_{i},q\right)+\beta \cdot s_{\mathrm{context}}\left(e_{i},q\right)+\gamma \cdot s_{\mathrm{recency}}\left(e_{i}\right)+\delta \cdot s_{\mathrm{importance}}\left(e_{i}\right),$$

where query $q=(\phi_{q},\kappa_{q})$ represents current compressed features and contextual information, and the weighting coefficients α=0.4, β=0.2, γ=0.2, δ=0.2 reflect the relative importance of different similarity aspects based on cognitive psychology principles. The weighting coefficients were determined through empirical validation on held-out data, balancing the contributions of different similarity aspects for optimal retrieval performance. Pattern similarity $s_{\mathrm{pattern}}\left(e_{i},q\right)$ employs cosine similarity between compressed feature representations:

(10) $$s_{\mathrm{pattern}}\left(e_{i},q\right)=\frac{\phi_{i}\cdot \phi_{q}}{∥\phi_{i}{∥}_{2}{∥\phi_{q}∥}_{2}}.$$

Contextual similarity $s_{\mathrm{context}}\left(e_{i},q\right)$ measures alignment between environmental and situational factors:

(11) $$s_{\mathrm{context}}\left(e_{i},q\right)=\frac{1}{\left|K\right|}\sum_{k\in K}\mathrm{sim}\left(\kappa_{i}^{\left(k\right)},\kappa_{q}^{\left(k\right)}\right),$$

where K represents the set of contextual features (brightness, contrast, spatial properties) and sim(·,·) computes normalized similarity for each contextual dimension. Recency boost $s_{\mathrm{recency}}\left(e_{i}\right)$ implements exponential decay that prioritizes recently formed memories:

(12) $$s_{\mathrm{recency}}\left(e_{i}\right)=\exp(-\frac{t_{\mathrm{current}}-t_{i}}{100}),$$

where $t_{\mathrm{current}}$ represents the current global time step and $t_{i}$ denotes the timestamp when experience $e_{i}$ was stored. Importance score $s_{\mathrm{importance}}\left(e_{i}\right)$ reflects the learned significance of each memory based on emotional valence, access frequency, and consolidation strength:

(13) $$s_{\mathrm{importance}}\left(e_{i}\right)=\frac{w_{\mathrm{emotion}}\left(e_{i}\right)+w_{\mathrm{access}}\left(e_{i}\right)+w_{\mathrm{novelty}}\left(e_{i}\right)}{3},$$

where emotional weighting $w_{\mathrm{emotion}}\left(e_{i}\right)\in \left[0.3,1.0\right]$ assigns higher importance to very positive (1.0) and very negative (0.9) experiences, access weighting $w_{\mathrm{access}}\left(e_{i}\right)$ increases with retrieval frequency following $w_{\mathrm{access}}\left(e_{i}\right)=1.0+0.1\cdot {\mathrm{access}\_\mathrm{count}}_{i}$, and novelty weighting captures the uniqueness of the stored pattern relative to the existing Memory Bank. The system retrieves top-k=9 most relevant experiences, which undergo integration through multi-head attention mechanisms. The memory attention mechanism enables pattern matching between current queries and historical experiences:

(14) $$A_{\mathrm{memory}}=\mathrm{MultiHead}\left(\phi_{q},\left\{\phi_{1},\ldots ,\phi_{k}\right\},\left\{\phi_{1},\ldots ,\phi_{k}\right\}\right),$$

where the multi-head attention employs 8 attention heads, each computing:

(15) $$\mathrm{Attention}\left(Q,K,V\right)=\mathrm{softmax}(\frac{QK^{T}}{\sqrt{d_{k}}})V,$$

with $Q=\phi_{q}W_{Q}$, $K=\left[\phi_{1},\ldots ,\phi_{k}\right]W_{K}$, and $V=\left[\phi_{1},\ldots ,\phi_{k}\right]W_{V}$, where $W_{Q},W_{K},W_{V}\in R^{128\times 16}$ are learned projection matrices for each attention head, and $d_{k}=16$ is the dimension per head. The attended memory representations are then fused with current query features through a learned combination:

(16) $$\phi_{\mathrm{enhanced}}=\Theta_{\mathrm{fusion}}\left(\mathrm{Cat}\left(\phi_{q},A_{\mathrm{memory}}\right)\right),$$

where $\Theta_{\mathrm{fusion}}:R^{256}\to R^{128}$ integrates current and historical information through a learned linear transformation, enabling the memory system to adaptively weight the contribution of recalled experiences based on their relevance to the current segmentation task.

#### 3.4.4. Memory-Guided Feature Enhancement

The Memory Enhancement component, as illustrated in Figure 2, modifies backbone features through learned projection of memory-derived insights. This process represents the culmination of memory system processing, where AEK directly influences current feature representations:

(17) $$F_{\mathrm{enhanced}}^{\left(3\right)}=F^{\left(3\right)}+\lambda \cdot \Theta_{\mathrm{influence}}\left(\phi_{\mathrm{enhanced}}\right)⊙1_{H_{3}\times W_{3}},$$

where $\Theta_{\mathrm{influence}}:R^{128}\to R^{1536}$ projects memory information to match backbone feature dimensions, λ=0.2 controls memory influence strength, and ⊙ represents element-wise multiplication broadcasted across spatial dimensions. This enables the memory system to provide contextual guidance that improves segmentation accuracy, particularly in challenging scenarios where current visual information alone may prove insufficient for reliable road area detection. The enhanced features then proceed through the standard UPerNet decoder processing, benefiting from memory-informed representations that capture learned associations and contextual patterns accumulated during training.

### 3.5. Training Strategy and Memory Dynamics

Our training methodology carefully orchestrates the dual objectives of accurate segmentation and effective memory formation through multi-objective optimization. The system operates in continuous learning mode, where each prediction contributes to both immediate performance evaluation and long-term knowledge accumulation. The training objective combines segmentation accuracy with memory utilization effectiveness:

(18) $$L_{\mathrm{total}}=L_{\mathrm{seg}}+\mu \cdot L_{\mathrm{memory}}.$$

Segmentation loss balances pixel-wise accuracy with boundary preservation through a weighted combination of Binary Cross-Entropy (BCE) and Dice formulations:

(19) $$L_{\mathrm{seg}}=0.4\cdot L_{\mathrm{BCE}}\left(\hat{Y},Y\right)+0.6\cdot L_{\mathrm{Dice}}\left(\hat{Y},Y\right),$$

where the BCE loss is computed as:

(20) $$L_{\mathrm{BCE}}\left(\hat{Y},Y\right)=-\frac{1}{N}\sum_{i=1}^{N}\left[Y_{i}\log\left(\sigma \left({\hat{Y}}_{i}\right)\right)+\left(1-Y_{i}\right)\log\left(1-\sigma \left({\hat{Y}}_{i}\right)\right)\right],$$

and the Dice loss encourages spatial coherence and is computed as:

(21) $$L_{\mathrm{Dice}}\left(\hat{Y},Y\right)=1-\frac{2\sum_{i=1}^{N}\sigma \left({\hat{Y}}_{i}\right)Y_{i}+\epsilon }{\sum_{i=1}^{N}\sigma \left({\hat{Y}}_{i}\right)+\sum_{i=1}^{N}Y_{i}+\epsilon }$$

where σ denotes the sigmoid activation and $\epsilon =1\times 10^{-6}$ provides numerical stability. Furthermore, memory regularization encourages effective utilization of retrieved experiences through explicit alignment between current query features and recalled memory representations:

(22) $$L_{\mathrm{memory}}=\frac{1}{K}\sum_{k=1}^{K}{∥\phi_{\mathrm{query}}-\phi_{\mathrm{memory}}^{\left(k\right)}∥}_{2}^{2},$$

where $\phi_{\mathrm{query}}\in R^{128}$ represents the current compressed query features, $\phi_{\mathrm{memory}}^{\left(k\right)}\in R^{128}$ denotes the *k*-th recalled memory pattern, and *K* is the number of retrieved memories (typically K=9). The memory regularization weight μ=0.1 balances segmentation accuracy with memory coherence, ensuring that retrieved experiences provide meaningful contextual guidance without overwhelming the primary segmentation objective. It promotes alignment between current features and recalled memories when such alignment benefits prediction accuracy, ensuring that memory retrieval mechanisms contribute meaningfully to segmentation performance.

Beyond basic storage and retrieval, our memory system implements dynamics that mirror human memory processes. The forgetting mechanism applies gradual decay with access-dependent preservation, ensuring that frequently accessed and important memories persist while less relevant experiences fade naturally:

(23) $$\iota_{t+1}^{\left(i\right)}=\iota_{t}^{\left(i\right)}\cdot \rho_{\mathrm{decay}}\cdot \left(1+0.1\cdot \alpha_{t}^{\left(i\right)}\right),$$

where $\rho_{\mathrm{decay}}=0.995$ represents the base decay rate, $\alpha_{t}^{\left(i\right)}$ counts access frequency, and $\iota_{t}^{\left(i\right)}$ denotes the importance score of memory *i* at time *t*. This implements a “use it or lose it” principle that maintains system efficiency while preserving valuable experiential knowledge. Memory consolidation occurs through sleep-like processes implemented at regular intervals during training, strengthening important memories while optimizing storage efficiency:

(24) $$\iota_{\mathrm{consolidated}}^{\left(i\right)}=\iota_{\mathrm{current}}^{\left(i\right)}\cdot \left\{\begin{array}{cc} 1.1 & \mathrm{if}\;\epsilon_{i}\in \left\{\mathrm{very}\_\mathrm{positive},\mathrm{very}\_\mathrm{negative}\right\}\\ 1.0 & \mathrm{otherwise} \end{array}\right.,$$

where emotional experiences receive importance boosts during consolidation, reflecting the psychological principle that emotionally significant events are preferentially retained in long-term memory. The Memory Bank capacity management employs intelligent forgetting based on comprehensive importance metrics rather than simple temporal ordering:

(25) $$F_{i}=\arg\min_{i}\left(\iota_{t}^{\left(i\right)}\cdot s_{\mathrm{recency}}\left(e_{i}\right)\cdot w_{\mathrm{emotion}}\left(e_{i}\right)\right),$$

where $F_{i}$ is the index of the item to be forgotten, ensuring that the least important memories are removed when capacity constraints require memory optimization. The training process employs progressive memory integration, beginning with reduced memory influence during early epochs and gradually increasing memory utilization as the system develops proficiency in both segmentation and memory management. This curriculum approach ensures stable convergence while encouraging memory-prediction interactions as training progresses.

## 4. Experiments and Results

This section presents a comprehensive experimental validation of the proposed framework. We demonstrate its effectiveness through systematic evaluation against state-of-the-art methodologies and detailed ablation studies examining individual component contributions.

### 4.1. Datasets

Virtual KITTI 2 [6] provides approximately 21,000 photo-realistic synthetic images spanning diverse weather conditions, lighting variations, and camera perspectives. This dataset reproduces five original KITTI sequences with comprehensive multimodal data, including RGB imagery, depth information, and precise semantic segmentation annotations, making it particularly valuable for memory system training due to its controlled environmental variations.

KITTI road [4] comprises 289 training and 290 test images captured through car-mounted stereo camera systems in real urban environments. This dataset is organized into three distinct categories: UM (Urban Marked) featuring 95 training and 96 test images of roads with clearly visible lane markings, UMM (Urban Multiple Marked lanes) containing 96 training and 94 test images depicting complex multi-lane scenarios with multiple lane markings, and UU (Urban Unmarked) encompassing 98 training and 100 test images of roads without visible lane markings. The dataset features high-resolution imagery with meticulously annotated ground-truth road/non-road binary masks for the training set, representing the gold standard benchmark for road area segmentation evaluation.

Cityscapes [3] encompasses approximately 5000 finely annotated images supplemented by 20,000 coarsely annotated samples across 50 European cities. This dataset provides pixel-level annotations for 30 distinct semantic classes, including detailed road surface annotations, offering extensive diversity in urban road scenarios for comprehensive generalization assessment.

R2D [41] offers synthetic imagery with precise road area annotations enhanced by surface normal information. This dataset complements real-world data with controlled synthetic scenarios, enabling systematic evaluation of MA approaches across varied environmental conditions.

### 4.2. Training and Testing Protocol

**Figure 4:**
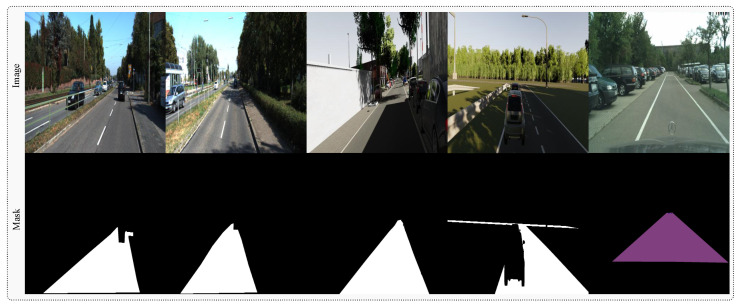
Representative samples illustrating the diversity of training data, including input images and corresponding ground-truth road area masks across various environmental conditions and road configurations. Zoom in for a better view.

We establish a unified training corpus by combining Virtual KITTI 2, KITTI road, Cityscapes, and R2D training images, creating a comprehensive dataset that balances synthetic diversity with real-world authenticity. This configuration enables our memory system to accumulate experiences across both controlled synthetic environments and authentic driving scenarios. Some samples from the datasets, along with images and their ground-truth masks, are shown in Figure 4. Evaluation employs the official KITTI road test set through provider-based evaluation protocols, supplemented by results on the Cityscapes and R2D datasets to assess generalization capabilities.

To enhance model robustness and facilitate effective memory formation across diverse scenarios, we employ comprehensive data augmentation strategies. These transformations include random horizontal flips with 50% probability, random rotations spanning $-15^{\circ }$ to $+15^{\circ }$, and color jittering with brightness, contrast, saturation, and hue adjustments up to 20%. Additionally, we apply geometric transformations including translations up to 10% of image dimensions and scaling factors ranging from 0.9 to 1.1, supplemented by random cropping and selective blurring with 30% probability. It should be noted that our framework processes only monocular RGB images as input, without utilizing stereo depth information or any additional modalities.

### 4.3. Experimental Setup

All experiments were conducted using the PyTorch version 2.9.0 deep-learning framework on a computational system equipped with 8 shared NVIDIA RTX A6000 GPUs (NVIDIA, Santa Clara, CA, USA) and 755 GB RAM, powered by 2 Intel Xeon Gold 6330 processors (Intel, Santa Clara, CA, USA) with 112 cores. The InternImage-XL backbone was initialized using official pretrained weights to leverage established feature representations, while our Memory Bank system was trained from scratch to develop domain-specific associative patterns. Hyperparameter configuration was carefully optimized for training, as shown in Table 1. The Adam optimizer was employed with an initial learning rate of $1\times 10^{-4}$, complemented by ReduceLROnPlateau scheduling with 2-epoch patience and 0.1 reduction factor. Our combined Dice and BCE loss formulation balanced pixel-wise accuracy with boundary preservation. Training employed a batch size of 2 to accommodate the computational overhead of memory operations, with input images resized to 640 × 640 pixels to preserve spatial detail essential for effective memory formation and retrieval.

We follow standard evaluation protocols using official test splits with fixed random seeds for reproducibility. The consistent performance across various datasets shows our method works reliably beyond single test results.

### 4.4. Evaluation Metrics

We adopt the standard evaluation protocol established by [4] for comprehensive performance assessment. Primary metrics include Precision (PRE), Average Precision (AP), Recall (REC), Intersection over Union (IoU), Accuracy (ACC), False Positive Rate (FPR), False Negative Rate (FNR), and Maximum F1-Score (MaxF) for quantitative segmentation quality evaluation. MaxF represents the Maximum F1-Score across all confidence thresholds and serves as the primary performance indicator following KITTI road benchmark conventions.

### 4.5. Comparison with State-of-the-Art Multimodal Methods

To assess the effectiveness of our framework, we conducted extensive comparisons with cutting-edge multimodal road segmentation methodologies. These approaches incorporate sensor fusion techniques, integrating diverse information sources including RGB imagery, LiDAR point clouds, surface normal estimations, and depth data to achieve superior segmentation performance through complementary modality exploitation.

Table 2 presents a comprehensive performance evaluation on the official KITTI road benchmark, demonstrating our framework’s competitive positioning within the established state-of-the-art landscape. Our approach achieves a MaxF score of 0.9666, placing it among the top-performing methodologies (on KITTI leaderboard) while operating exclusively on single-modality, i.e., RGB input only. The framework exhibits particularly robust precision (0.9395), indicating excellent discriminative capabilities that effectively minimize false road predictions, a critical requirement for autonomous driving safety.

The comparative analysis reveals that despite operating without the informational advantages of multimodal sensor fusion, our approach maintains performance levels that are remarkably competitive with leveraging LiDAR, depth, and surface normal information. This achievement underscores the effectiveness of MA inference in compensating for single-modality constraints through intelligent utilization of AEK. The slightly elevated false positive and false negative rates reflect the inherent challenges of single-modality processing, yet the overall performance demonstrates that memory mechanisms can effectively bridge the information gap typically addressed through sensor diversity. While multimodal approaches achieve marginally superior performance, they require complex sensor suites, sophisticated calibration procedures, and substantial computational resources that may not be practical for all autonomous vehicle configurations.

**Figure 5:**
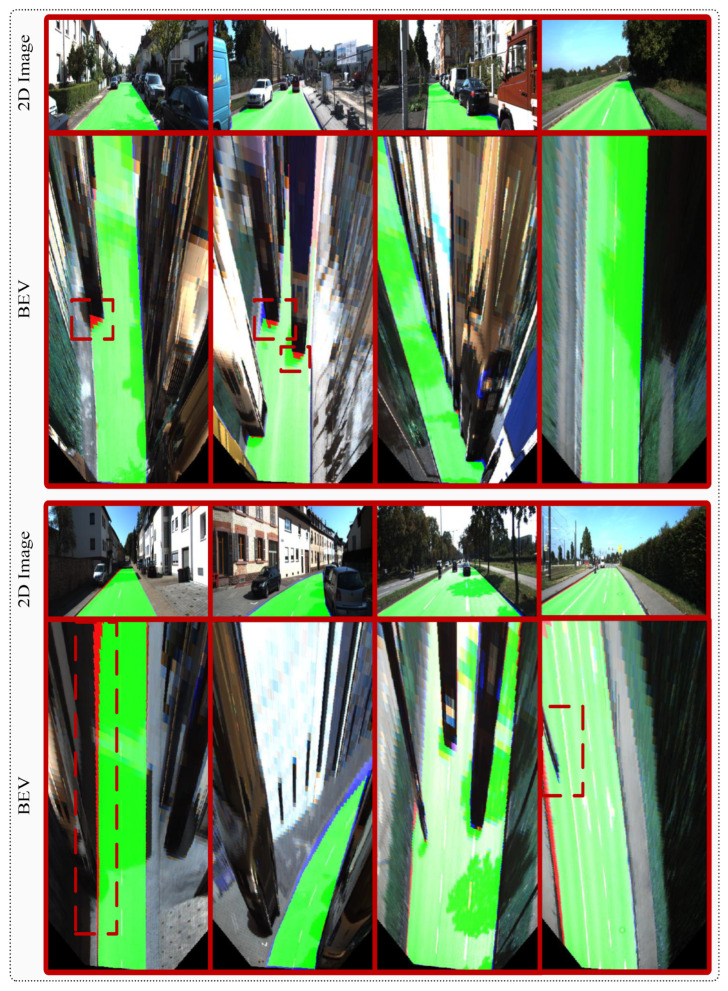
Evaluation visualization on the KITTI road dataset showing perspective view results (odd rows) and corresponding Bird’s Eye View (BEV) analysis (even rows). The color coding represents segmentation quality: green areas indicate true positives (correctly identified road regions), red areas denote false negatives (missed road areas), and blue areas correspond to false positives (incorrectly classified road regions). The BEV representation provides a comprehensive spatial assessment of segmentation accuracy across diverse road geometries and environmental conditions. Zoom in for a better view.

Figure 5 provides a detailed visual analysis of our framework’s performance on the KITTI road benchmark, demonstrating segmentation quality through both perspective and Bird’s Eye View (BEV) representations. The comprehensive evaluation reveals that our approach achieves robust road detection across diverse scenarios, with minimal false negatives (red regions) and well-controlled false positives (blue regions), while maintaining excellent true positive coverage (green regions) that accurately delineates drivable road areas.

### 4.6. Comparison with State-of-the-Art Single-Modality Methods

To provide a comprehensive evaluation context, we conducted a detailed comparison with leading single-modality road segmentation approaches. Our method shows competitive performance through architectural innovations and algorithmic optimizations.

The single-modality comparison presented in Table 3 reveals that our framework achieves notable performance advantages over existing RGB-only approaches. With a MaxF score of 0.9666, our method substantially outperforms the leading single-modality approaches, including RBANet (0.9630) and LC-CRF (0.9568), while demonstrating strong precision capabilities (0.9395) that exceed most compared methodologies.

This performance comparison highlights the transformative impact of memory augmentation in single-modality processing. Traditional RGB-only approaches are fundamentally constrained by the limited information available in individual frames, requiring architectural innovations to achieve competitive performance. Our framework transcends these limitations by accumulating and leveraging experiential knowledge from previously encountered scenarios, effectively expanding the informational context available for segmentation decisions. Furthermore, the competitive precision achieved by our approach indicates that memory-guided inference provides valuable contextual discrimination that helps distinguish genuine road areas from ambiguous regions that might confuse traditional feed-forward processing. This capability is particularly valuable in challenging scenarios, including shadowed road surfaces, complex geometric configurations, and partial occlusions, where accumulated experiential patterns can provide decisive contextual guidance. Additionally, our framework demonstrates strong recall performance while maintaining competitive precision compared with single-modality approaches, indicating a favorable balance between comprehensive road detection and false positive minimization. This characteristic is essential for autonomous driving applications where both missed road areas and incorrectly identified obstacles can compromise navigation safety.

In order to provide a better understanding, Table 4 shows the performance of our framework across different KITTI road categories. The results for the KITTI dataset are calculated using the official evaluation server. The results can be viewed at the official website (https://www.cvlibs.net/datasets/kitti/eval_road_detail.php?result=d9e4ae6781d5c6dbf01d5799bfbf1665afd89a8b accessed 19 October 2025). Furthermore, Table 4 presents the official precision, recall curves over various categories.

**Figure 6:**
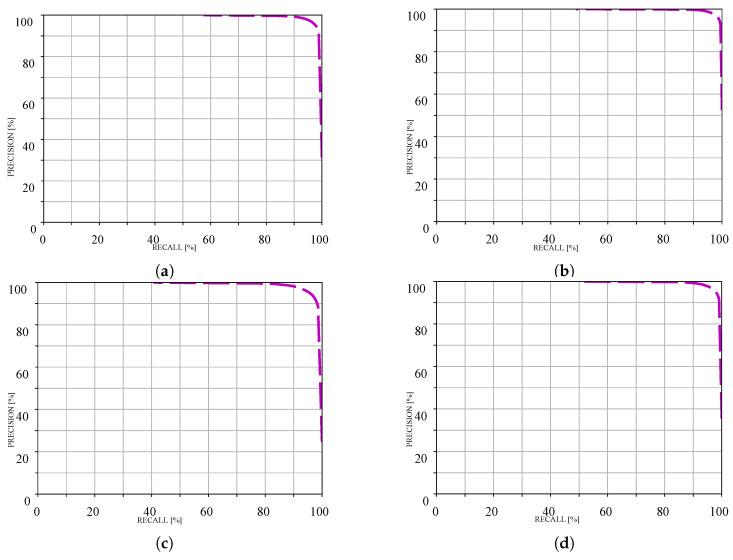
Precision-recall curves across KITTI road categories (computed by official evaluation server): (**a**) UM road, (**b**) UMM road, (**c**) UU road, and (**d**) URBAN road.

To provide better insights to the readers, the official precision and recall curves are presented in Figure 6.

### 4.7. Comprehensive Ablation Studies

We conducted systematic ablation studies to evaluate the individual contributions of different memory system components and design choices. These experiments were performed on a carefully curated evaluation subset combining Virtual KITTI 2 and KITTI road datasets, enabling controlled assessment of memory dynamics and their impact on segmentation performance.

#### 4.7.1. Impact of Memory System Integration

**Figure 7:**
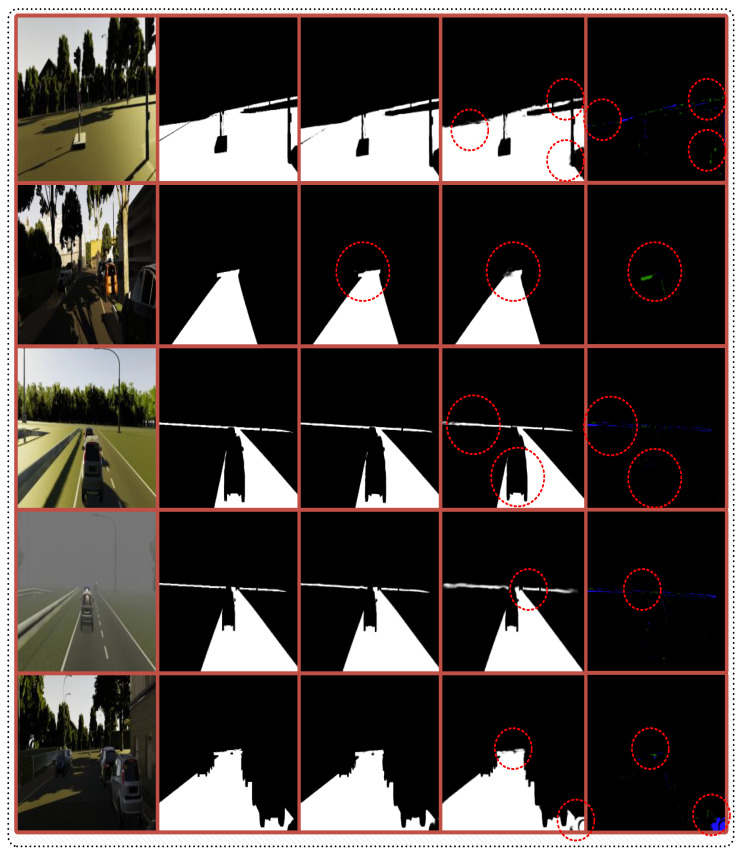
Qualitative comparison of MA versus baseline segmentation performance. From left: input images, ground-truth, MA predictions, baseline predictions, and difference analysis. Green regions indicate successful road recovery through memory guidance, blue regions show false positive reduction, and black regions represent comparable performance. Zoom in for a better view.

Figure 7 demonstrates the practical effectiveness of memory augmentation through direct visual comparison across challenging road scenarios.

The comparative analysis reveals that memory integration provides substantial improvements in challenging scenarios. Green-highlighted regions demonstrate successful recovery of road segments missed by the baseline approach, particularly in complex geometric configurations and shadowed areas. Blue regions indicate reduced false positives through AEK, while black regions show comparable performance between approaches. Memory augmentation proves especially effective in ambiguous boundary conditions where visual information alone proves insufficient. The system leverages accumulated contextual associations to distinguish genuine road areas from challenging background regions, demonstrating the practical value of experiential knowledge accumulation in spatial reasoning tasks.

#### 4.7.2. Memory Bank Capacity Analysis

Memory capacity analysis reveals that larger memory banks provide enhanced performance through increased diversity of stored experiences. The 200-memory configuration achieves an optimal balance between experiential coverage and computational efficiency, enabling comprehensive knowledge retention while preserving real-time processing capabilities essential for autonomous driving applications. The progressive improvement with increased capacity demonstrates the memory system’s ability to leverage richer experiential knowledge for enhanced segmentation accuracy.

#### 4.7.3. Memory Influence Weight Optimization

Memory weight analysis demonstrates that a stronger memory influence (λ=0.5) yields optimal performance with a MaxF of 0.9907 and exceptional average precision of 0.9995. This configuration effectively balances current visual information with AEK, enabling the memory system to provide substantial contextual guidance while preserving the network’s ability to process novel scenarios. The reduced false positive rate (0.0024) at higher memory weights indicates that accumulated experiences help discriminate challenging road boundaries more effectively.

#### 4.7.4. Loss Function Component Analysis

Loss function analysis in Table 5 reveals that Binary Cross-Entropy (BCE) achieves superior individual performance (for our method), while the combined BCE+Dice formulation provides balanced optimization for both pixel-wise accuracy and boundary preservation. The combined approach demonstrates robust performance across all metrics, making it optimal for MA training where both precise classification and sharp boundary delineation are essential for effective experiential knowledge accumulation.

#### 4.7.5. Pretrained Weight Initialization Impact

Pretrained weight initialization analysis in Table 6 indicates that both approaches achieve comparable performance, with training from scratch showing marginal advantages in recall and false negative rate. This suggests that the MA framework can effectively develop domain-specific representations regardless of initialization strategy, highlighting the robustness of the memory-guided learning process in adapting to road segmentation tasks.

### 4.8. Performance on R2D

The R2D dataset evaluation examines our framework’s effectiveness on synthetic road scenarios enhanced with surface normal information, providing a controlled assessment of memory-guided segmentation across varied geometric configurations and lighting conditions.

The R2D evaluation results presented in Table 7 demonstrate our framework’s robust cross-domain adaptation capabilities. Achieving a MaxF score of 0.9490 with particularly strong precision, our approach maintains competitive performance despite the domain shift from real-world KITTI training data to synthetic R2D scenarios. This performance indicates that the AEK successfully generalizes across different visual characteristics and geometric configurations. The memory system’s effectiveness in synthetic environments suggests that the learned associative patterns capture fundamental road segmentation principles that transcend specific imaging conditions. The competitive precision performance (0.9545) compared with several established multimodal approaches highlights the Memory Bank’s discriminative capabilities in distinguishing genuine road areas from challenging background regions, even when encountering novel synthetic visual characteristics not present in the original training distribution.

### 4.9. Performance on Cityscapes

Cityscapes evaluation provides a comprehensive assessment of memory-guided segmentation across diverse European urban environments, examining the framework’s adaptability to varied architectural styles, road configurations, and metropolitan driving scenarios.

The Cityscapes evaluation results demonstrated in Table 8 reveal compelling evidence of our memory system’s robust generalization across diverse metropolitan environments. Achieving a MaxF score of 0.9189 with comparable recall performance, our framework successfully adapts to the complex urban road configurations characteristic of European cities while maintaining competitive positioning among established methodologies. The competing recall performance indicates that our approach excels at comprehensive road area detection across the varied geometric configurations, intersection patterns, and architectural contexts present in Cityscapes imagery. This capability suggests that AEK effectively captures generalizable road segmentation patterns that transcend specific geographical and architectural characteristics, enabling robust performance across diverse urban environments.

### 4.10. Computational Efficiency Analysis

Our framework achieves a favorable performance-efficiency trade-off. While requiring additional memory operations and computational resources (358 M parameters, 476 G FLOPs), the framework achieves near state-of-the-art performance through intelligent utilization of accumulated experiences. Compared with top-performing methods such as SNE-RoadSeg (1950.2G FLOPs) and PLARD (1147.6G FLOPs), our approach demonstrates competitive segmentation quality, making it suitable for autonomous driving applications where both accuracy and resource considerations are important.

Real-time performance is crucial for autonomous driving applications. Our framework achieves an average inference speed of 18.5 FPS on a shared NVIDIA RTX A6000 GPU for 640 × 640 input images. The memory system’s computational overhead remains acceptable for real-time deployment, as the quality improvements justify the speed reduction compared with baseline approaches.

### 4.11. Qualitative Analysis

**Figure 8:**
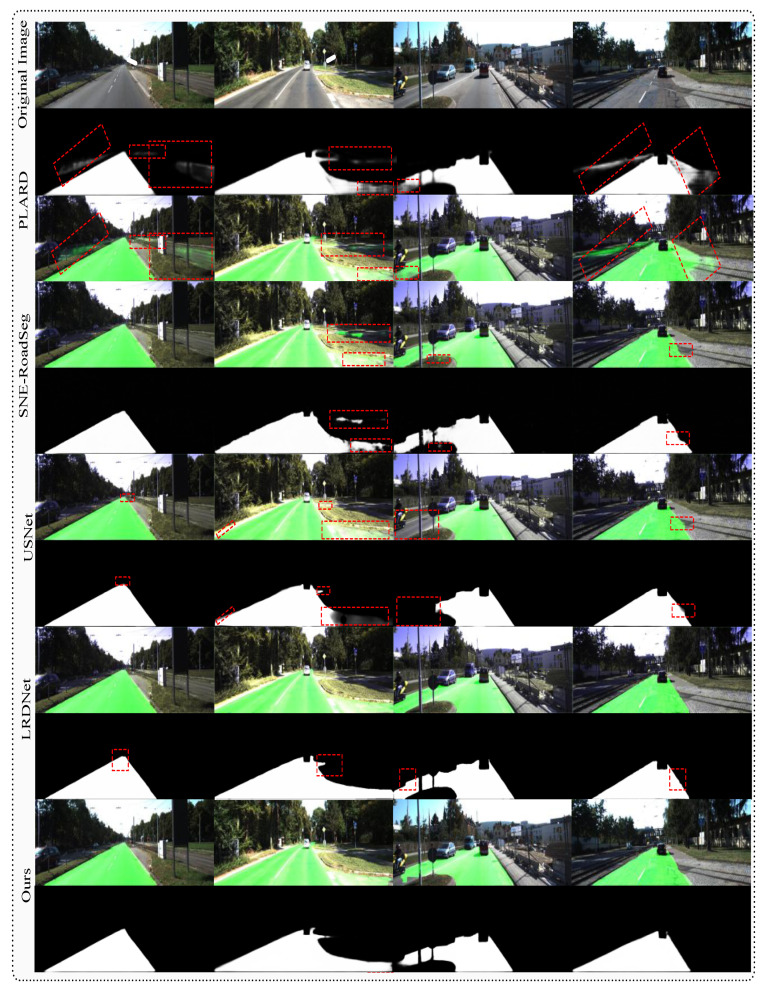
Qualitative comparison of road segmentation results from various state-of-the-art methods on the KITTI road dataset. Green regions indicate predicted drivable areas, while red boxes highlight areas of segmentation difficulty or failure. Our approach demonstrates robust performance across diverse road scenarios. Zoom in for a better view.

Figure 8 presents a qualitative comparison of road segmentation results demonstrating the effectiveness of MA inference across challenging scenarios. The memory system particularly excels in complex road geometries, shadowed regions, and ambiguous boundary conditions where AEK provides valuable contextual guidance for accurate segmentation decisions.

The qualitative results reveal that memory augmentation enhances segmentation consistency and boundary precision, particularly in challenging scenarios where traditional feed-forward processing encounters ambiguity. The memory system’s ability to recall relevant experiential patterns enables more confident and accurate predictions in complex driving environments.

## 5. Limitations, Environmental Impact, and Future Directions

While our framework demonstrates competitive performance, several limitations warrant discussion. The current implementation achieves 18.5 FPS on an NVIDIA RTX A6000 GPU. This may fall short of ultra-high-speed requirements for certain applications. Evaluation on embedded automotive-grade hardware (e.g., NVIDIA Xavier, Orin) would provide valuable deployment insights. However, consistent with other state-of-the-art methods in the literature, our results are reported on non-embedded GPU platforms. The memory system introduces computational overhead. However, this remains competitive with other high-performing methods such as SNE-RoadSeg and PLARD, as shown in Table 9.

The fixed memory capacity (200 experiences) with importance-based forgetting may introduce catastrophic forgetting risks during extended deployment when encountering continuously diverse scenarios. The current mechanism may inadvertently discard valuable rare-event patterns crucial for handling edge cases. Future work will investigate replay-based consolidation strategies and hierarchical memory architectures to address this limitation.

Framework performance depends on several hyperparameters, including memory size, influence weight (λ), and top-*k* retrieval count. While ablation studies (Table 5, Table 10 and Table 11) demonstrate robustness across tested configurations, optimal settings may vary across different deployment scenarios. The emotional valence categorization relies on fixed IoU thresholds. More sophisticated adaptive quality metrics could improve memory formation effectiveness.

Generalization to significantly different real-world conditions beyond our training distribution remains challenging. Our training data primarily covers public datasets. Performance may degrade when encountering extreme weather conditions, unpaved rural roads, or novel infrastructure designs not present in training data. Future research will explore domain adaptation techniques and active learning strategies to improve robustness across diverse operational conditions.

For ${\mathrm{CO}}_{2}$ emissions, training our model for 100 epochs produces approximately 12–15 kg ${\mathrm{CO}}_{2}$ equivalent (assuming 0.429 kg ${\mathrm{CO}}_{2}$/kWh grid intensity [42]).

Future work could explore memory compression and efficient retrieval mechanisms. Adaptive capacity management could address memory constraints. Finally, investigating meta-learning approaches for automated hyperparameter optimization would enable self-tuning memory systems capable of adjusting to diverse operational conditions.

## 6. Conclusions

In this paper, we present a free road space detection framework that integrates human-inspired cognitive architectures with deep-learning models for enhanced image feature extraction, contributing to the advancement of sensor-based computer vision systems. The proposed Human-like Memory Bank system implements episodic, semantic, and working memory subsystems with biologically inspired consolidation and forgetting mechanisms. We demonstrate the effectiveness of experiential knowledge accumulation in improving detection performance. Experimental results indicate that our single-modality RGB approach achieves superior performance among all single-modality methods and competitive performance approaching state-of-the-art multimodal systems through intelligent memory utilization. The comprehensive ablation studies confirm the individual contributions of different memory components. The framework’s ability to maintain this competitive performance while operating exclusively on RGB input demonstrates a favorable performance-efficiency trade-off compared with methods with significantly higher computational requirements, effectively narrowing the gap between single-modality and multimodal approaches. Future research directions include extending memory mechanisms to multi-task learning scenarios, investigating adaptive memory consolidation strategies, and exploring applications to other computer vision tasks requiring contextual reasoning and experiential knowledge accumulation.

## Figures and Tables

**Table 1:** Hyperparameter settings for training.

Parameter	Value
Optimizer	Adam
Learning rate	$1.00\times 10^{-4}$
LR scheduler	ReduceLROnPlateau
Scheduler patience	2
Scheduler factor	0.1
Memory weight	0.2
Loss function	Combined (Dice + BCE)
Epochs	100
Batch size	2
Image size	640 × 640
Memory size	200
Top-*k* memories	9

**Table 2:** Performance comparison with state-of-the-art multimodal road segmentation methods on official KITTI road test set. (↑) indicates higher values are preferred, while (↓) signifies lower values are optimal. Results are arranged in descending order of performance based on the official KITTI leaderboard, with our method listed in the last row.

Method	MaxF (↑)	AP (↑)	PRE (↑)	REC (↑)	FPR (↓)	FNR (↓)
DiPFormer [22]	0.9757	0.9294	0.9734	0.9779	0.0147	0.0221
RoadFormer+ [24]	0.9756	0.9374	0.9743	0.9769	0.0142	0.0231
SNE-RoadSegV2 [21]	0.9755	0.9398	0.9757	0.9753	0.0134	0.0247
UdeerLID+ [30]	0.9755	0.9398	0.9746	0.9765	0.0140	0.0235
RoadFormer [23]	0.9750	0.9385	0.9716	0.9784	0.0157	0.0216
SNE-RoadSeg+ [20]	0.9750	0.9398	0.9741	0.9758	0.0143	0.0242
Pseudo-LiDAR [27]	0.9742	0.9409	0.9730	0.9754	0.0149	0.0246
Evi-RoadSeg [15]	0.9708	0.9354	0.9657	0.9759	0.0191	0.0241
PLARD [18]	0.9703	0.9403	0.9719	0.9688	0.0154	0.0312
LRDNet+ [14]	0.9695	0.9222	0.9688	0.9702	0.0172	0.0298
USNet [19]	0.9689	0.9325	0.9651	0.9727	0.0194	0.0273
LRDNet (L) [14]	0.9687	0.9191	0.9673	0.9701	0.0181	0.0299
DFM-RTFNet [25]	0.9678	0.9405	0.9662	0.9693	0.0187	0.0307
SNE-RoadSeg [41]	0.9675	0.9407	0.9690	0.9661	0.0170	0.0339
LRDNet(S) [14]	0.9674	0.9254	0.9679	0.9669	0.0176	0.0331
3MT-RoadSeg [26]	0.9660	0.9390	0.9646	0.9673	0.0195	0.0327
TEDNet [28]	0.9462	0.9305	0.9428	0.9496	0.0317	0.0504
CLRD [29]	0.9420	0.9266	0.9425	0.9414	0.0316	0.0586
CLCFNet [17]	0.9638	0.9085	0.9638	0.9639	0.0199	0.0361
LFD-RoadSeg [16]	0.9521	0.9371	0.9535	0.9508	0.0256	0.0492
Ours	0.9666	0.9395	0.9646	0.9687	0.0196	0.0313

**Table 3:** Comparison with state-of-the-art single-modality road segmentation methods on the official KITTI road test set. Results are presented in descending order according to performance on the official KITTI leaderboard, with our method listed in the final row.

Method	MaxF (↑)	AP (↑)	PRE (↑)	REC (↑)	FPR (↓)	FNR (↓)
RBANet [36]	0.9630	0.8972	0.9514	0.9750	0.0275	0.0250
CLCFNet (LiDAR) [17]	0.9597	0.9061	0.9612	0.9582	0.0213	0.0418
LC-CRF [31]	0.9568	0.8834	0.9362	0.9783	0.0367	0.0217
Hadamard-FCN [37]	0.9485	0.9148	0.9481	0.9489	0.0286	0.0511
HA-DeepLabv3+ [35]	0.9483	0.9324	0.9477	0.9489	0.0288	0.0511
DEEP-DIG [34]	0.9398	0.9365	0.9426	0.9369	0.0314	0.0631
LFD-RoadSeg [16]	0.9349	0.9219	0.9346	0.9352	0.0213	0.0648
RoadNet3 [32]	0.9295	0.9193	0.9332	0.9258	0.0216	0.0742
ChipNet [33]	0.9291	0.8495	0.9098	0.9491	0.0306	0.0509
Ours	0.9666	0.9395	0.9646	0.9687	0.0196	0.0313

(↑) indicates that higher values are better; (↓) indicates that lower values are better.

**Table 4:** Performance comparison of the KITTI official evaluation across categories.

Benchmark	MaxF (↑)	AP (↑)	PRE (↑)	REC (↑)	FPR (↓)	FNR (↓)
UM road	0.9655	0.9358	0.9646	0.9664	0.0161	0.0336
UMM road	0.9746	0.9557	0.9707	0.9786	0.0325	0.0214
UU road	0.9537	0.9276	0.9508	0.9566	0.0161	0.0434
Urban road	0.9666	0.9395	0.9646	0.9687	0.0196	0.0313

(↑) indicates that higher values are better; (↓) indicates that lower values are better.

**Table 5:** Comparative analysis of different loss function formulations on MA training effectiveness.

Loss Function	MaxF (↑)	AP (↑)	PRE (↑)	REC (↑)	FPR (↓)	FNR (↓)
BCE loss	0.9905	0.9995	0.9920	0.9891	0.0024	0.0109
Dice loss	0.9873	0.9941	0.9880	0.9866	0.0036	0.0134
Combined (BCE + Dice)	0.9905	0.9995	0.9912	0.9898	0.0026	0.0102

(↑) indicates that higher values are better; (↓) indicates that lower values are better.

**Table 6:** Evaluation of pretrained weight initialization versus training from scratch on the MA framework performance.

Initialization	MaxF (↑)	AP (↑)	PRE (↑)	REC (↑)	FPR (↓)	FNR (↓)
Training from scratch	0.9896	0.9991	0.9905	0.9887	0.0028	0.0113
Pretrained InternImage	0.9893	0.9992	0.9908	0.9878	0.0027	0.0122

(↑) indicates that higher values are better; (↓) indicates that lower values are better.

**Table 7:** Comprehensive performance comparison on the R2D dataset demonstrating cross-domain generalization capabilities of our approach versus established methodologies.

Method	MaxF (↑)	PRE (↑)	REC (↑)
SNE-RoadSeg [41]	0.9505	0.9450	0.9561
LRDNet+ [14]	0.9459	0.9382	0.9538
LRDNet (L) [14]	0.9406	0.9462	0.9350
LRDNet (S) [14]	0.9373	0.9325	0.9421
USNet [19]	0.9366	0.9310	0.9423
RBANet [36]	0.9329	0.9354	0.9305
DFM-RTFNet [25]	0.9298	0.9275	0.9321
3MT-RoadSeg [26]	0.9287	0.9312	0.9263
TEDNet [28]	0.9156	0.9089	0.9225
CLCFNet [17]	0.9145	0.9201	0.9090
Ours	0.9490	0.9545	0.9436

(↑) indicates that higher values are better.

**Table 8:** Detailed performance analysis on the Cityscapes dataset revealing memory system effectiveness across diverse urban road scenarios and architectural environments.

Method	MaxF (↑)	PRE (↑)	REC (↑)
SNE-RoadSeg [41]	0.9275	0.9290	0.9261
USNet [19]	0.9269	0.9201	0.9337
LRDNet+ [14]	0.9265	0.9228	0.9302
LRDNet (L) [14]	0.9247	0.9098	0.9401
HA-DeepLabv3+ [35]	0.9233	0.9277	0.9189
LRDNet (S) [14]	0.9176	0.8805	0.9580
DFM-RTFNet [25]	0.9134	0.9156	0.9112
3MT-RoadSeg [26]	0.9089	0.9123	0.9055
RBANet [36]	0.8982	0.9014	0.8950
TEDNet [28]	0.8945	0.8976	0.8914
CLCFNet [17]	0.8923	0.8845	0.9003
CLRD [29]	0.8867	0.8901	0.8834
Ours	0.9189	0.9259	0.9120

(↑) indicates that higher values are better.

**Table 9:** Computational efficiency comparison in terms of parameters and floating-point operations.

Model	Params. (M)	FLOPs (G)
LRDNet+	28.5	336
LRDNet (L)	19.5	173
SNE-RoadSeg	201.3	1950.2
USNet	30.7	78.2
PLARD	76.9	1147.6
RBANet	42.1	156.8
Ours	358	476

**Table 10:** Investigation of memory influence strength on segmentation performance, revealing optimal weighting for memory-guided feature enhancement.

Memory Weight	MaxF (↑)	AP (↑)	PRE (↑)	REC (↑)	FPR (↓)	FNR (↓)
λ=0.1	0.9905	0.9992	0.9914	0.9896	0.0026	0.0104
λ=0.3	0.9900	0.9991	0.9908	0.9892	0.0027	0.0108
λ=0.5	0.9907	0.9995	0.9918	0.9895	0.0024	0.0105

(↑) indicates that higher values are better; (↓) indicates that lower values are better.

**Table 11:** Performance sensitivity analysis across different Memory Bank capacities, demonstrating optimal size selection for balancing experiential coverage.

Memory Size	MaxF (↑)	AP (↑)	PRE (↑)	REC (↑)	FPR (↓)	FNR (↓)
50 memories	0.9896	0.9994	0.9910	0.9882	0.0027	0.0118
100 memories	0.9893	0.9994	0.9904	0.9882	0.0029	0.0118
200 memories	0.9899	0.9993	0.9911	0.9886	0.0026	0.0114

(↑) indicates that higher values are better; (↓) indicates that lower values are better.

## Data Availability

Training and evaluation are conducted on publicly available datasets (KITTI road, Cityscapes, Virtual KITTI 2, R2D) with standard evaluation protocols. The code, weights, and other materials can be found at https://github.com/abdkhanstd/MemRoadNet (accessed on 19 October 2025).
